# Susceptibility of Chinese Perch Brain (CPB) Cell and Mandarin Fish to Red-Spotted Grouper Nervous Necrosis Virus (RGNNV) Infection

**DOI:** 10.3390/ijms17050740

**Published:** 2016-05-19

**Authors:** Jiagang Tu, Wenjie Chen, Xiaozhe Fu, Qiang Lin, Ouqin Chang, Lijuan Zhao, Jiangfeng Lan, Ningqiu Li, Li Lin

**Affiliations:** 1 Department of Aquatic Animal Medicine, College of Fisheries, Huazhong Agricultural University, Wuhan 430070, Hubei, China; tujiagang@mail.hzau.edu.cn (J.T.); chenwenjie53@163.com (W.C.); zhaolijuan4234@163.com (L.Z.); 2 Pearl River Fisheries Research Institute, Chinese Academy of Fishery Sciences, Key Laboratory of Fishery Drug Development, Ministry of Agriculture, Key Laboratory of Aquatic Animal Immune Technology, Guangzhou 510380, Guangdong, China; fuxiaozhe-1998@163.com (X.F.); lin9902057@163.com (Q.L.); chang-ouqin@126.com (O.C.)

**Keywords:** Mandarin fish, NNV, CPB, brain cell line, susceptibility

## Abstract

Nervous necrosis virus (NNV) is the causative agent of viral encephalopathy and retinopathy (VER), a neurological disease responsible for high mortality of fish species worldwide. Taking advantage of our established Chinese perch brain (CPB) cell line derived from brain tissues of Mandarin fish (*Siniperca chuatsi*), the susceptibility of CPB cell to Red-Spotted Grouper nervous necrosis virus (RGNNV) was evaluated. The results showed that RGNNV replicated well in CPB cells, resulting in cellular apoptosis. Moreover, the susceptibility of Mandarin fish to RGNNV was also evaluated. Abnormal swimming was observed in RGNNV-infected Mandarin fish. In addition, the cellular vacuolation and viral particles were also observed in brain tissues of RGNNV-infected Mandarin fish by Hematoxylin-eosin staining or electronic microscopy. The established RGNNV susceptible brain cell line from freshwater fish will pave a new way for the study of the pathogenicity and replication of NNV in the future.

## 1. Introduction

Nervous necrosis virus (NNV), or called betanodavirus, is a non-enveloped icosahedral RNA virus that belongs to the family of *Nodaviridae*. Its genome composed of two single-stranded positive-sense RNA molecules (RNA1 and RNA2) [1]. The RNA1 (3.1 Kb) encodes RNA-dependent RNA polymerase, and RNA2 (1.4 Kb) encodes capsid protein [1]. In addition, a subgenomic RNA3 that is transcribed from RNA1 segment encodes the non-structural B1 and B2 proteins [2,3,4,5,6]. Betanodaviruses can be classified into four genotypes based on the analysis of RNA2 segment [7,8]. Among them, Red-Spotted Grouper nervous necrosis virus (RGNNV) was the widest spread genotype.

The infection of betanodaviruses is usually characterized by abnormal swimming associated with vacuolation and necrosis in the central nervous tissues and retina [9]. Therefore, the disease was named viral nervous necrosis (VNN) or viral encephalopathy and retinopathy (VER). To date, several fish cell lines have been shown to be susceptible to NNV [10,11,12,13,14,15,16]. However, due to the lack of susceptible cell lines derived from fish nervous tissues, the neurotrophy of NNV remains enigmatic. Therefore, it is highly expected to establish a NNV-susceptible cell line derived from fish nervous tissues.

NNV was initially isolated from diseased marine fish and has been reported in more than 30 commercial marine fish species all over the world [9]. Recently, increasing evidence shows that NNV has also been epidemic in freshwater fish [17,18,19,20,21]. In addition, experimental infections also revealed that some freshwater fish were susceptible to NNV [22,23]. NNV usually infects the larvae or juvenile of fish, but evidence has also indicated that NNV can infect mature groupers (*Epinephelus* spp.), resulting in high mortality [8]. Belonging to the same family (*Serranidae*) with groupers, Mandarin fish (*Siniperca chuatsi*), as an economically important species, attracted our attentions for its susceptibility to NNV. Therefore, in this study, the Chinese perch brain (CPB) cell derived from brain tissues of Mandarin fish was used to evaluate its susceptibility to RGNNV as well as the cellular apoptosis induced by RGNNV infection. Then the experimental infection of RGNNV in Mandarin fish was further performed to confirm the susceptibility *in vivo*. Histopathological features of the RGNNV-infected Mandarin fish were also examined by either H and E staining or electronic microscopy. The results obtained here might shed a new light on the host range and infectivity of betanodaviruses.

## 2. Results

### 2.1. CPB Cells Are Susceptible to RGNNV

**Figure 1:**
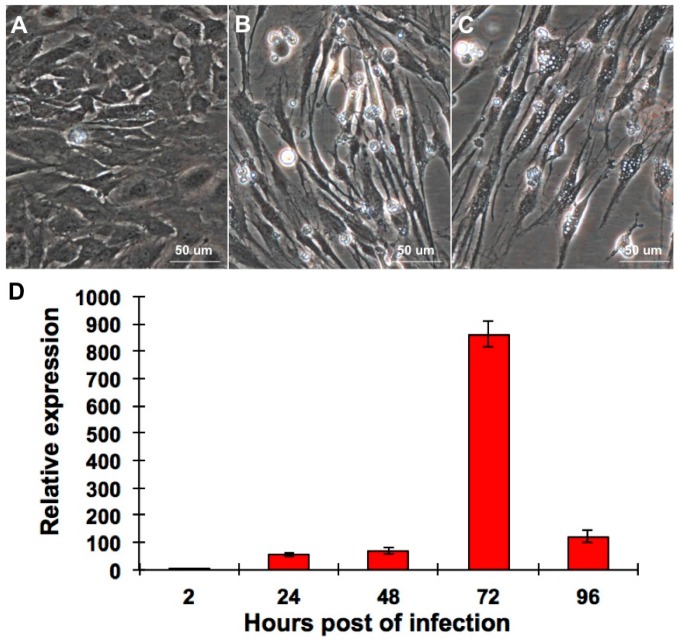
(**A**) Mock-infected CPB cells; (**B**) RGNNV-infected CPB cells at three days p.i.; (**C**) RGNNV-infected CPB cells at seven days p.i.; (**D**) The qRT-PCR was performed to quantify RGNNV replication in CPB cells. CPB cells were infected with 0.01 MOI of RGNNV and the relative expression of capsid protein gene of RGNNV was detected at different time point p.i. β-actin was used as internal reference gene.

The CPB cells were infected with 0.01 multiplicity of infection (MOI) of RGNNV to evaluate its susceptibility to RGNNV. Compared with mock-infected CPB cells, dead cells were observed in RGNNV-infected CPB cells at three and seven days post infection (p.i.) (Figure 1A–C). Meanwhile, The quantitative reverse transcription polymerization chain reaction (qRT-PCR) was performed to evaluate RGNNV replication in CPB cells. As shown in Figure 1D, the relative particle number of RGNNV increased until 72 h p.i. and decreased then, indicating that RGNNV replicated well in CPB cells.

### 2.2. RGNNV Infection Induced Apoptosis in CPB Cells

**Figure 2:**
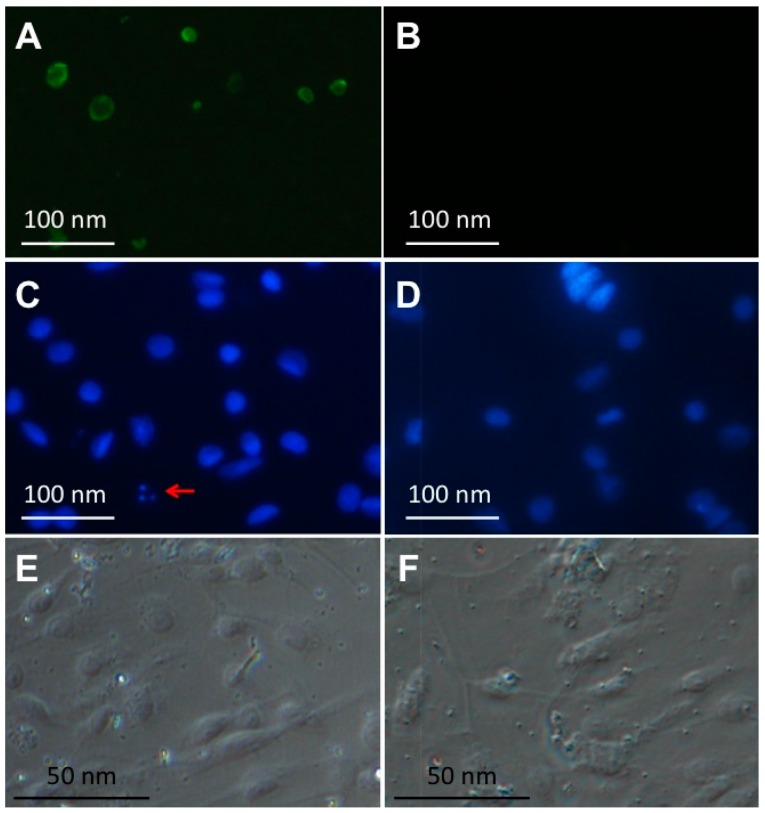
(**A**) Cellular apoptosis detection in RGNNV-infected CPB cells using annexin V-FITC/PI staining. Green fluorescence represents early apoptosis; (**B**) Cellular apoptosis detection in mock-infected CPB cells using annexin V-FITC/PI staining. (**C**–**F**) Cellular apoptosis of RGNNV-infected CPB cells (**C**,**E**) and mock-infected CPB cells (**D**,**F**) was measured using DAPI. The red arrow in (**C**) represents apoptotic bodies.

Annexin V-FITC/propidium iodide (PI) double staining was used to determine the cellular apoptosis of CPB cells when infected with 1 MOI of RGNNV (Figure 2A,B). The basic principle of this method was described previously [24]. During the early stage of apoptosis, the phospholipids on inner side of cellular membrane were transported to the outer side and thus bound with FITC-labeled annexin V, while during the late stage of apoptosis, a fluorescent molecule PI can bind to DNA and result in the excitation of red fluorescence. In this study, when mock-infected CPB cells were treated with annexin V/PI double staining, there was no green signal (Figure 2B). However, green but not red signal was observed in RGNNV-infected CPB cells at 24 h p.i. (Figure 2A), suggesting that RGNNV infection caused apoptosis at an early stage. Furthermore, the nuclei of CPB cells were stained with DAPI in order to confirm that RGNNV infection could induce cellular apoptosis (Figure 2C–F). The apoptotic bodies were observed in RGNNV-infected CPB cells at 48 h p.i. (Figure 2C), but not in mock-infected cells (Figure 2D). Taken together, RGNNV infection could induce cellular apoptosis in CPB cells.

### 2.3. Clinical Signs of Mandarin Fish upon RGNNV Infection

**Figure 3:**
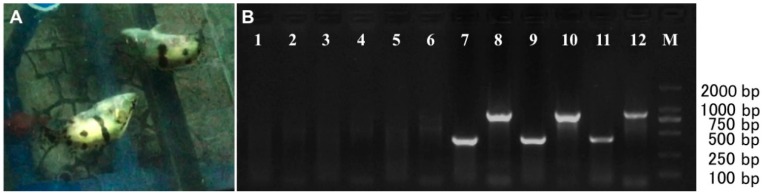
(**A**) The symptom of abnormal swimming of RGNNV-infected Mandarin fish; (**B**) detection of RGNNV in the brains of diseased and non-infected control Mandarin fish. Amplification of the T2 domain (875 bp) and T4 domain (426 bp) of capsid protein gene of RGNNV from brains of three mock-infected (lane 1–6) and RGNNV-infected (lane 7–12) Mandarin fish; M: marker.

To further investigate the susceptibility of brain tissues of Mandarin fish to RGNNV, RGNNV was injected into the Mandarin fish via eyeball. At four days p.i., abnormal swimming behavior was observed from two of the five RGNNV-infected fish (Figure 3A and Supplementary Material). All five RGNNV-infected fish exhibited abnormal swimming behavior at five days p.i. However, mock-infected fish swam normally during the observation period. The brain tissues from three randomly selected RGNNV-infected or mock-infected Mandarin fish at five days p.i. were subjected to an RT-PCR assay with the detection of two different domains of capsid protein gene (Figure 3B). RGNNV was detected in brain tissues of all three RGNNV-infected Mandarin fish, but not from the brain tissues of mock-infected Mandarin fish (Figure 3B).

### 2.4. Histopathology of Mandarin Fish Infected with RGNNV

**Figure 4:**
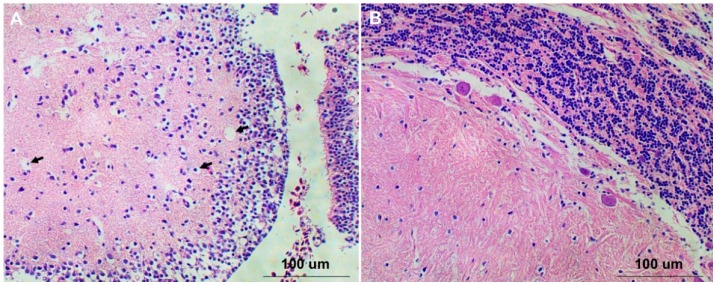
(**A**) The brain tissue of RGNNV-infected Mandarin fish. Black arrows pointed to vacuoles; (**B**) The brain tissue of mock-infected Mandarin fish.

The brain tissues from RGNNV-infected and mock-infected Mandarin fish were sampled for histological examination. No apparent pathogenic changes were observed in brain tissues from mock-infected fish (Figure 4B), while numerous vacuoles were observed in brain tissues of RGNNV-infected fish (Figure 4A), indicating that RGNNV can infect the brain tissues of Mandarin fish through the eyeball injection route and induce vacuolation to brain tissues.

### 2.5. Visualization of RGNNV Particles in Brain Tissues of RGNNV-Infected Mandarin Fish

**Figure 5:**
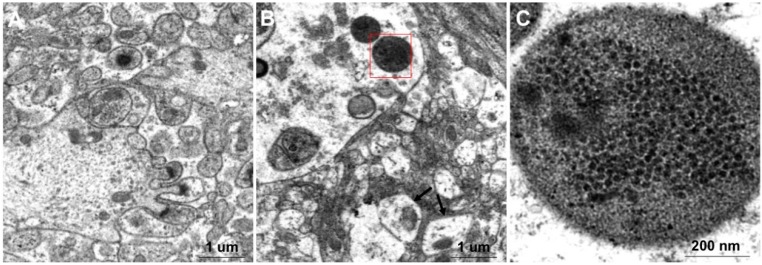
Observation of the negatively stained brain tissues from RGNNV-infected and mock-infected Mandarin fish using electron microscopy. (**A**) Brain tissue of mock-infected mandarin fish; (**B**) Brain tissue of RGNNV-infected mandarin fish. Black arrows indicate that mitochondria are swollen; (**C**) Enlarged view from (**B**) shows that a lot of closely arranged particles were enclosed in the inclusion body.

The mock-infected and RGNNV-infected brain tissues were observed under electronic microscope. The mitochondria of brain cells from RGNNV-infected fish were swollen (Figure 5B) compared to those from mock-infected fish (Figure 5A). A lot of ~30 nm particles closely arranged in the inclusion body were observed in cytoplasm of the brain cells from RGNNV-infected fish (Figure 5B,C). The size and shape of these particles were identical to RGNNV particles, further indicating that brain tissue of Mandarin fish is susceptible to RGNNV infection.

## 3. Discussion

Many cell lines have been established to evaluate their susceptibility to NNV during the past two decades, including SSN-1 cells from *Channa striata* [10], GF-1 cells from *Epinephelus coioides* [25], SF and SB cells from *Lates calcarifer* [26,27], GB cells from *Epinephelus awoara* [11], and GS cells from *Epinephelus coioides* [28]. Among these cell lines, SSN-1 cells have been widely used for NNV infection, but the replication of NNV in SSN-1 cells was low and not stable [10]. Therefore, E-11 cells were subcloned from SSN-1 cells and have been shown to better support the replication of NNV [29,30]. However, the tissue origin of E-11 cells was not known. GB cells were the only cell line derived from brain tissue, which was from a marine fish grouper [11]. In this study, CPB cell that was derived from brain tissues of a freshwater Mandarin fish was chosen to evaluate its susceptibility to RGNNV. Consistent with the observation of other susceptible cell lines upon NNV infection, numerous vacuoles were observed in RGNNV-infected CPB cells.

NNV was initially isolated from marine fish. However, outbreaks caused by NNV have been reported recently in freshwater fish [17,18,19,20,21]. In addition, some freshwater fish were reported susceptible to NNV under experimental infection conditions [22,23], indicating a potential spread of NNV in freshwater fish. NNV usually causes mass mortality to the larvae or juvenile of fish, but for groupers, even the grow-out stage was highly susceptible to NNV infection [8]. Therefore, Mandarin fish, which are phylogenetically close to groupers, was attractive to evaluate their susceptibility to NNV. Different infection routes including immersion, cohabitation, intramuscular injection, and intraperitoneal injection have been commonly used to study the susceptibility of fish to *Betanodavirus* [31,32,33]. In this study, we first tried intraperitoneal injection route to evaluate the susceptibility of Mandarin fish to RGNNV. However, no abnormal clinical signs were observed even at 15 days p.i. (data not shown). Therefore, the eyeball injection route was used, as it was reasonable to believe that it might be a better way to deliver viruses to a place close to the brain. Neurological signs with abnormal swimming behavior were observed in Mandarin fish from four days p.i., indicating that eyeball injection was an efficient way for RGNNV infection. Except for the abnormal swimming behavior, vacuolation was observed in RGNNV-infected brains from Mandarin fish, which was consistent with the observation in NNV-infected brains from other fish species [19,34,35,36]. Meanwhile, we have also injected the RGNNV into snakehead fish (the host of SSN-1 cells) via the eyeball, no clinical sign was observed (data not shown), indicating that the infection of Mandarin fish was not an artifact of eyeball infection. In fact, fish with injured eyeballs are sometimes observed in cultured or wild-ranged ones, implying that the injury of eyeballs might also be a way for the transmission of NNV.

Betanodavirus can induce host cellular death and post-apoptotic necrosis in fish cells [37]. Non-structural protein B2 of betanodavirus was reported as an inducer of mitochondria-mediated cell death [5]. 

## 4. Material and Methods

### 4.1. Cells and Virus

The CPB cell, which was established from brain tissues of Mandarin fish [24], was cultured in M199 medium (Invitrogen, Carlsbad, CA, USA) supplemented with 10% fetal bovine serum (FBS) at 28 °C with 5% CO_2_. RGNNV was isolated from brains of diseased red-spotted grouper and kept in our laboratory [38]. In brief, brain tis*s*ues of diseased Red-Spotted Grouper were homogenized in PBS (1 g/mL) supplemented with 100 µg/mL penicillin and streptomycin. Homogenate was frozen and thawed three times and then centrifuged at 3000× *g* for 10 min, and the resulting supernatants were used to detect RGNNV using RT-PCR. The supernatants with RGNNV were stored at −80 °C for later use.

### 4.2. CPB Cell Infection

About 80% confluent CPB cells were infected with 0.01 MOI of RGNNV. After 2 h adsorption at 28 °C, the inoculum was removed and the cells were washed twice with PBS followed by adding M199 medium with 5% FBS. At three and seven days p.i., the CPB cells were imaged by an inverted fluorescence microscope (Nikon, Tokyo, Japan).

### 4.3. Virus RT-PCR and qRT-PCR Assays

Total RNA was isolated from tissues using TRIzol reagent (Invitrogen) and then subjected to cDNA synthesis. 1 µL of RNA was added to 19 µL of RT reaction mix containing 4 µL of first strand buffer, 1 µL of 20 µM reverse primer oligo dT, 8 µL of 2.5 mM dNTP mixture, 0.2 µL of RNase inhibitor (Invitrogen), 0.4 µL of M-MLV (Promega, Madison, WI, USA), and 5.4 µL of DEPC-treated water. The mixture was incubated at 42 °C for 30 min followed by 75 °C for 10 min. The synthesized cDNAs were used as templates for PCR amplification using the primers [39]. PCR amplification was carried out in a volume of 20 µL containing 10 µL Premix Ex Taq (Takara, Dalian, China), 1 µL each forward and reverse primers (10 mM), 7 µL nuclease-free water, and 1 µL cDNA. Cycling parameters were 94 °C for 5 min, followed by 35 cycles of 94 °C for 30 s, 55 °C for 30 s, and 72 °C for 30 s, one cycle at 72 °C for 10 min, and finally incubation at 4 °C for 10 min. The expected target amplified PCR products by these primers were 875 bp (T2) and 426 bp (T4). The PCR products were subjected to electrophoresis in 1% agarose gel.

QRT-PCR was used to quantify RGNNV replication in CPB cells. The PrimeScript RT reagent kit with gDNA Eraser (Takara, Dalian, China) was used for cDNA synthesis according to the manufacturer’s protocol. The quantitative PCR reactions were conducted in 20 µL volumes containing 10 µL of the SYBR Master Mix, 1 µL diluted cDNA, and 300 nM of each primer. The cycling conditions of quantitative PCR reactions were described as previously [24]. The β–actin gene was used as control. Amplification results were analyzed using a comparative Ct method as described before [24].

### 4.4. Investigation of Apoptosis in RGNNV-Infected CPB Cells

CPB cells at six-well plates were infected with 1 MOI of the RGNNV isolated from diseased red-spotted grouper or equal volume of PBS as negative control. At 24 h p.i., cells were used for apoptosis assay using annexin V-FITC/PI apoptosis detection kit (TransGen Biotech, Beijing, China) as described previously [24]. For DAPI staining, cells were treated as described before [24]. Finally the cells were viewed using an inverted fluorescence microscope.

### 4.5. Infection of Mandarin Fish with RGNNV

Mandarin fish with mean body length of 12 cm were purchased from a farm in Guangzhou city, China. Fish were maintained at 28 ± 0.5 °C in a recirculating freshwater system and were fed with forage fish for at least two weeks so that they acclimated to the laboratory conditions before experiments. Forage fish were fingerlings of smelt fish (*Osmerus mordax*). Forage fish and Mandarin fish were free of RGNNV, as tested by RT-PCR. Mandarin fish were anaesthetized by tricaine methanesul-A fonate (MS-222) according to the standard protocol recommended by the manufacturer before challenge. Experiments were approved by the guidelines of Institutional Animal and Care and Use Committees (IACUC) of Huazhong Agricultural University. The fish were divided randomly into two groups with five fish each. One group of fish was injected through the eyeball with 200 µL of 1:100 dilution of the supernatant with RGNNV from brain tissues of diseased Red-Spotted Grouper, while the second group was injected with equal volume of PBS. Clinical signs were recorded daily for two weeks. The brains of diseased and control fish were homogenized to detect RGNNV using RT-PCR, fixed in 4% paraformaldehyde for hematoxylin and eosin staining, or fixed in 2.5% glutaraldehyde for electron microscopic observation.

## 5. Conclusions

In this study, two methods including annexin V-FITC/PI double staining and DAPI staining were used and confirmed that RGNNV infection can induce apoptosis in CPB cells. It has been shown that apoptosis is controlled at the mitochondrial level and correlated with loss of mitochondrial membrane potential in NNV-infected fish cells [37]. Electronic microscopy showed that the mitochondria of brain cells from RGNNV-infected Mandarin fish were swollen compared with those from mock-infected fish (Figure 5B). Whether this phenomenon associated with the loss of mitochondrial membrane potential needs further study. The establishment of the fish brain originated CPB cell line, which was susceptible to NNV infection, will facilitate the study of the replication and pathogenicity of NNV in the future.

## Supplementary Materials

Supplementary materials can be found at http://www.mdpi.com/1422-0067/17/5/740/s1.
